# Insulin and Memory in Invertebrates

**DOI:** 10.3389/fnbeh.2022.882932

**Published:** 2022-04-26

**Authors:** Junko Nakai, Nozomi Chikamoto, Kanta Fujimoto, Yuki Totani, Dai Hatakeyama, Varvara E. Dyakonova, Etsuro Ito

**Affiliations:** ^1^Department of Biology, Waseda University, Tokyo, Japan; ^2^Laboratory of Biochemistry, Faculty of Pharmaceutical Sciences, Tokushima Bunri University, Tokushima, Japan; ^3^Koltzov Institute of Developmental Biology, Russian Academy of Sciences, Moscow, Russia; ^4^Graduate Institute of Medicine, School of Medicine, Kaohsiung Medical University, Kaohsiung, Taiwan

**Keywords:** *Caenorhabditis elegans*, classical conditioning, *Drosophila*, insulin-like growth factor, memory, *Lymnaea*, starvation, insulin

## Abstract

Insulin and insulin-like peptides (ILP) help to maintain glucose homeostasis, whereas insulin-like growth factor (IGF) promotes the growth and differentiation of cells in both vertebrates and invertebrates. It is sometimes difficult to distinguish between ILP and IGF in invertebrates, however, because in some cases ILP has the same function as IGF. In the present review, therefore, we refer to these peptides as ILP/IGF signaling (IIS) in invertebrates, and discuss the role of IIS in memory formation after classical conditioning in invertebrates. In the arthropod *Drosophila melanogaster*, IIS is involved in aversive olfactory memory, and in the nematode *Caenorhabditis elegans*, IIS controls appetitive/aversive response to NaCl depending on the duration of starvation. In the mollusk *Lymnaea stagnalis*, IIS has a critical role in conditioned taste aversion. Insulin in mammals is also known to play an important role in cognitive function, and many studies in humans have focused on insulin as a potential treatment for Alzheimer’s disease. Although analyses of tissue and cellular levels have progressed in mammals, the molecular mechanisms, such as transcriptional and translational levels, of IIS function in cognition have been far advanced in studies using invertebrates. We anticipate that the present review will help to pave the way for studying the effects of insulin, ILPs, and IGFs in cognitive function across phyla.

## Introduction

In 1988, a News and Views article, titled “Invertebrate neuroendocrinology. Insulin found at last?”, was published in *Nature* (Thorpe and Duve, 1988). This article introduced another report published in the same issue by Smit and colleagues from Vrije Universiteit, Amsterdam, describing the discovery of an insulin-related peptide in the central nervous system (CNS) of the gastropod mollusk *Lymnaea stagnalis* with convincing evidence that the functionally important peptide structure was conserved through a long period of evolution (Smit et al., 1988). The sequence data of this insulin-related peptide showed the presence of cysteines at positions in the A and B chains typical for the insulin family, suggesting the formation of an insulin-like tertiary structure with conserved (or alternative) hydrophobic core residues.

The background regarding the “first” insulin discovered in invertebrates, however, is somewhat complicated. In 1986 (2 years ahead of the study by Smit et al., 1988), Nagasawa et al. (1986) determined the complete amino acid sequence of 4K-PTTH-II, one of the three forms of the prothoracicotropic hormone, i.e., insulin-like peptide (ILP), of the silkworm *Bombyx mori*. 4K-PTTH-II is made up of two nonidentical peptide chains (A and B chains). This peptide has considerable sequence homology (40%) with human insulin, and the identical distribution of the six cysteine residues also indicates that 4K-PTTH-II belongs to the “insulin family”. Therefore, the definitive structural information on ILPs was obtained in *Bombyx mori* in 1986, but the first ILP DNA sequence was identified in *Lymnaea stagnalis* in 1988. In any case, these two studies together accelerated the functional analysis of ILPs in invertebrates (Ebberink et al., 1989).

In both vertebrates and invertebrates, not only do ILPs, including insulin, and insulin-like growth factors (IGFs) have similar structures (Blundell et al., 1978; Rinderknecht and Humbel, 1978; Smit et al., 1998; Brogiolo et al., 2001; Pierce et al., 2001; Li et al., 2003; Garelli et al., 2012), but the receptors to which they bind also have similar structures (Fernandez et al., 1995; Roovers et al., 1995; Kimura et al., 1997). The receptors for ILP and IGF have a structure that penetrates the cell membrane, with a binding site for ILP and IGF on the outside of the cell membrane. The receptor is a preformed, disulfide-bridged dimer. The ligand binding is thought to induce a conformational change in the receptor that initiates trans-phosphorylation of the two cytoplasmic domains and induces tyrosine kinase activity, thereby phosphorylating various substrates in the cell, which triggers downstream signals that result in a cellular response. With regard to insulin in mammals, the intracellular signaling mechanism by which the metabolism of glucose is regulated is well clarified (Saltiel and Kahn, 2001). IGF signals to cells that sufficient nutrients are available for cells to undergo hypertrophy and cell division, and these signals inhibit cell apoptosis and increase the production of cellular proteins (Shimizu, 2021). Although the signal transduction of IGF has been elucidated in terms of its commonalities with insulin, little is known about how the long-lasting cellular response caused by IGF is triggered (Andoh, 2021).

In invertebrates, many ILPs have also specific growth-promoting roles (Kenyon, 2010). For example, *Lymnaea* ILP, named “molluscan insulin-related peptide (MIP)”, was first identified as the product of cells noted for their role in controlling growth in invertebrates (Smit et al., 1988). *Lymnaea* MIPs also have a role in reducing the hemolymph glucose level (Mita et al., 2014a). It is therefore sometimes difficult to discriminate between invertebrate ILPs and IGFs. In the present article, we refer to ILP and IGF together as “ILP/IGF signaling (IIS)” in invertebrates.

IIS plays an important role in cognitive function in both vertebrates and invertebrates (Lin et al., 2010; Chen et al., 2011; Chambers et al., 2015). In mammals, especially humans, insulin is mostly investigated in relation to diabetes. Nasal administration of insulin is successfully used as a treatment for Alzheimer’s disease, and type-2 diabetes is associated with the risk of developing Alzheimer’s disease (Reger et al., 2008; Craft et al., 2012; Akinola, 2016). Insulin is not thought to pass through the blood-brain barrier, but it can be transported from the blood into the brain *via* receptors on vascular endothelial cells, which is called a receptor-mediated transporter pathway (Rhea and Banks, 2019). The effectiveness of insulin for Alzheimer’s disease may be explained from the viewpoint of metabolic stress (Wakabayashi et al., 2019) as follows: metabolic stress causes the onset of insulin resistance and lowers the removal rate of amyloid β proteins from the brain, thereby promoting their accumulation. Accordingly, insulin has been emphasized as a therapeutic drug for Alzheimer’s disease.

Insulin receptors are widely expressed in the brain of vertebrates, and insulin signaling regulates energy metabolism in the hypothalamus, motor function in the cerebellum, memory control, and nerve regeneration in the hippocampus, and emotional and cognitive function control in the cerebral cortex (Arnold et al., 2018). Administration of insulin into the ventricles of rats promotes Akt-dependent translocation of glucose transporter type 4 (GLUT4) in the hippocampus (Grillo et al., 2009). Furthermore, hippocampal-specific suppression of insulin signaling reduces long-term potentiation in the hippocampus and significantly impairs memory and learning ability (Grillo et al., 2015). As mentioned above, type 2 diabetes is associated with an increased risk for Alzheimer’s disease and impaired cognitive function (sometimes referred to as type 3 diabetes Steen et al., 2005). Insulin is a useful treatment, and thus many studies using mammalian brains have focused on the effectiveness of insulin against this disease. The molecular and cellular events behind the therapeutic effect of insulin, however, remain unknown. On the other hand, studies of the involvement of IIS in learning and memory have been performed in invertebrates. We therefore suggest that the studies using invertebrates to elucidate the mechanisms of IIS are particularly important for clarifying the function of insulin in cognition.

In the present review, we outline the studies of IIS mechanisms of memory regulation performed in three well-established invertebrate neuroscience model organisms, *Drosophila melanogaster*, *Caenorhabditis elegans*, and *Lymnaea stagnalis*. To the best of our knowledge, the molecular mechanisms of memory influenced by IIS function are not yet clarified in invertebrates other than these three species, even in the silkworm *Bombyx mori*. We will present the deduced pathways of IIS for memory by considering the experimental results in these three species, which may help to gain insight into the general scheme of the relationship between IIS function and memory.

## IIS and Memory in *Drosophila melanogaster*

**Figure 1:**
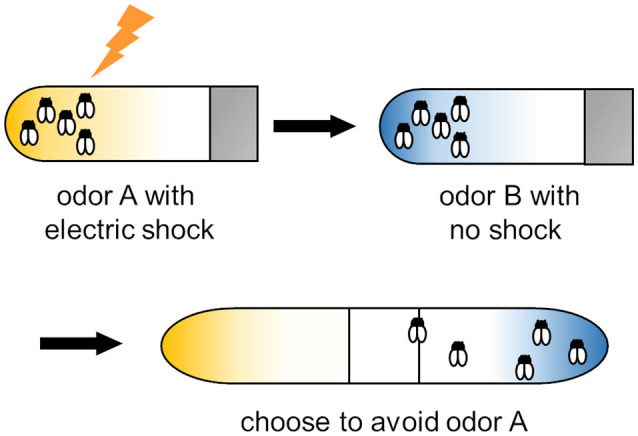
Training protocol for olfactory associative learning in *Drosophila*. Flies were exposed to odor A with an electric shock and then to odor B with no shock. After training, flies are placed at the choice point of a T maze with odor A or B diffused from both ends. Trained flies avoid the shock-paired odor (i.e., odor A).

In *Drosophila*, IIS is involved in memory formation, circadian rhythm formation, growth, development, reproduction, metabolism, stress resistance, aging, and lifespan (Barber et al., 2016; Semaniuk et al., 2021). IIS is required for both short-term memory and long-term memory of olfactory associative learning (Figure 1; Chambers et al., 2015). The main molecules of IIS—ILPs (Dilps), insulin receptor (InR), and insulin receptor substrate (CHICO)—are well studied in mutants. InR and CHICO are required for short-term memory based on studies of *chico* mutants (Naganos et al., 2012), for intermediate-term memory-based on studies of *InR* mutants (Tanabe et al., 2017), and for long-term memory based on studies of RNA interference for *inR* and *chico* (Chambers et al., 2015). *Drosophila* has eight types of Dilps (Nässel et al., 2013, 2015; Nässel and Vanden Broeck, 2016). In addition to binding to the receptor tyrosine kinase InR, Dilps can also interact with G protein-coupled receptors. For example, Dilp7 and Dilp8 activate the relaxin receptor-like LGR4 and LGR3, respectively (Colombani et al., 2015; Gontijo and Garelli, 2018; Mita, 2019; Veenstra et al., 2021; Veenstra, 2021). Moreover, Dilp3 functions in intermediate-term memory of aversive olfactory conditioning (Tanabe et al., 2017). In aged flies, a Dilp3 deficit causes memory loss.

The insulin receptor, InR, is involved in protein synthesis-independent “larval” anesthesia-resistant memory (lARM; Eschment et al., 2020). InR expressed in the mushroom body Kenyon cells suppresses lARM formation and contributes to the formation of protein synthesis-dependent longer-lasting memory in *Drosophila* larvae.

Mutant studies have demonstrated that the insulin receptor substrate CHICO plays an important role in olfactory associative learning (Naganos et al., 2012). CHICO was found to be essential for the development of a CNS region required for olfactory associative learning. CHICO also functions in food-finding latency (Egenriether et al., 2015). The food-finding latency differs between young and aged flies, with a shorter latency in aged flies. Even in young flies, however, the latency becomes shorter if they are starved or if the insulin signaling is reduced in *chico* mutants. Conversely, the latency of aged flies becomes longer when insulin signaling is enhanced. Learning deficiencies in *chico* mutants are due to a decrease in cAMP signaling (Naganos et al., 2016).

In 2013, a relationship between starvation and food aversive learning was demonstrated by Hirano and Saitoe (Hirano et al., 2013; Hirano and Saitoe, 2013). When cAMP-response element-binding protein (CREB) binds to cAMP-regulated transcriptional coactivator (CRTC), CREB upregulates some genes (Yin et al., 1994, 1995). CRTC is activated when insulin signaling is downregulated in starved flies (Wang et al., 2008). Thus, starved flies exhibit food aversive learning in one trial because of downregulation of the insulin signaling pathway and upregulation of the CRTC signaling pathway. Using *chico* mutants, Hirano et al. (2013) found that CRTC accumulated in the mushroom body nuclei (i.e., neurons necessary for memory), and reported that this mutant exhibited 1-trial learning even when satiated.

## IIS and Memory in *Caenorhabditis elegans*

IIS in *Caenorhabditis elegans* is involved in longevity, stress tolerance, and memory formation (Kenyon et al., 1993; Paradis et al., 1999; Stein and Murphy, 2012; Sasakura and Mori, 2013). The main components of the *C. elegans* IIS pathway include INS-1 (1 of 40 ILPs), DAF-2 (insulin receptor), AGE-1 (phosphoinositide 3-kinase: PI3K), DAF-16 (forkhead box protein O: FOXO), DAF-18 (phosphatase and tensin homolog deleted on chromosome 10: PTEN), and AKT-1 (serine/threonine kinase; Paradis et al., 1999; Pierce et al., 2001; Li et al., 2003; Tomioka et al., 2006; Murphy and Hu, 2013; Kaletsky et al., 2016; Kim and Webb, 2017). Several reports have demonstrated a relationship between IIS and memory in *C. elegans*. In IIS mutants, the declines with aging in isothermal tracking, motor activity, and positive butanone associative learning were suppressed, whereas the defects occurred in salt chemotaxis learning and benzaldehyde-starvation avoidance learning (Murphy and Hu, 2013). IIS controls salt chemotactic learning due to two types of changes, an increased Ca^2+^ response and decreased synaptic release, in the properties of a salt-sensing neuron, ASER, after prolonged NaCl exposure (Oda et al., 2011). A neuropeptide encoded by an insulin-like gene, *ins-11*, negatively regulates neuronal signaling that controls avoidance behavior toward a pathogen (*Pseudomonas aeruginosa*; Lee and Mylonakis, 2017).

**Figure 2:**
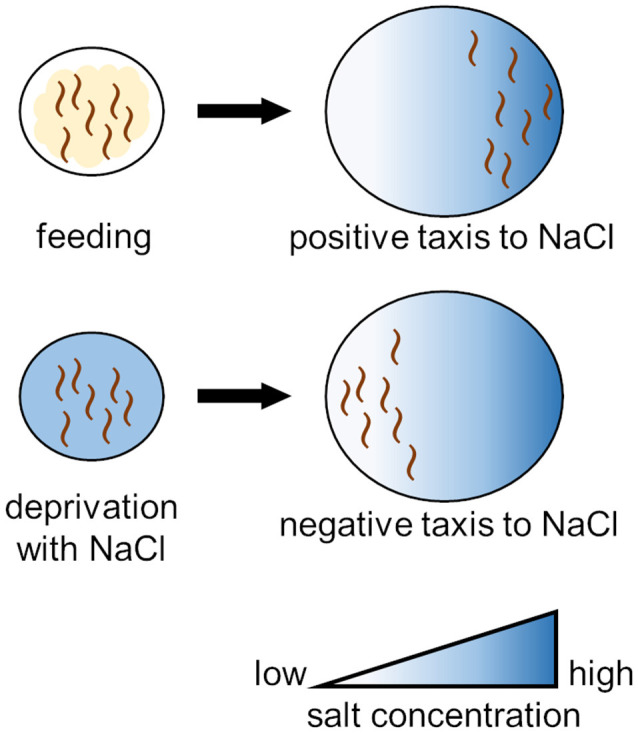
Training protocol for salt chemotaxis learning in *C. elegans*. Worms exhibit positive chemotaxis (attraction behavior) to NaCl under normal conditions, whereas worms subjected to prolonged exposure to NaCl under starvation conditions exhibit negative chemotaxis (avoidance behavior) to NaCl.

Nematodes grown under abundant food conditions show positive taxis (i.e., attraction behavior) to NaCl, whereas they show negative taxis (i.e., an avoidance behavior) to NaCl when grown under conditions of starvation but in the presence of NaCl. This is called salt chemotaxis learning (Figure 2; Saeki et al., 2001). This behavioral change does not occur in the case of starvation in the absence of NaCl or in the case of satiation in the presence of NaCl. In other words, when nematodes approach salt and fail to obtain food, they learn to avoid salt. In this learning, INS-1, DAF-2, and AGE-1 in the IIS pathway play important roles. These molecules function during the adult stage of *C. elegans* development after completion of the neural circuit formation. Nematodes deficient in INS-1 do not acquire this learning (Kodama et al., 2006; Oda et al., 2011). Ohno et al. (2014) reported that activation of the INS-1/DAF-2/PI3K pathway in a chemosensory neuron, ASER, altered the neuronal characteristics; e.g., a change in synaptic vesicle release from ASER. This associative learning was enhanced by IIS. DAF-2 has two splicing isoforms (DAF-2a and DAF-2c), and DAF-2c was found to function in taste-avoidance learning (Tomioka et al., 2022). Nagashima et al. (2019) demonstrated that the DAF-2c-dependent pathway acts in parallel with DAF-16.

IIS is important not only for learning by NaCl stimulation but also for learning by odor (benzaldehyde) stimulation (Lin et al., 2010). The dramatic change in odorant preference of a nematode after exposure to a high concentration of benzaldehyde in the absence of food is a result of associative learning in which the nematode forms an association between benzaldehyde and starvation (Nuttley et al., 2002). This behavioral plasticity is called benzaldehyde–starvation associative plasticity. Lin et al. (2010) demonstrated that nematodes with mutations in components of IIS pathways are defective in benzaldehyde-starvation associative plasticity and that IIS plays a more significant role in memory retrieval than in memory acquisition. INS-1 can act on multiple neurons and AGE-1 acts on benzaldehyde-sensing amphid wing C (AWC) sensory neurons to direct benzaldehyde–starvation associative plasticity. That is, these findings dissociate the behavioral roles of IIS in the regulation of learning vs. memory recall and help to elucidate the molecular mechanisms involved in this associative plasticity in *C. elegans*.

On the other hand, in associative learning between presentations of temperature and starvation, INS-1 acts on an interneuron involved in isothermal tracking behavior, antagonizes DAF-2 and AGE-1 and evokes food-related thermotactic plasticity (Murakami et al., 2005, Murakami, 2007). This learning ability declines with age. The age-related decay in *age-1* and *daf-2* mutants, both of which exhibit increased DAF-16 activity, is delayed. These effects are counteracted in *daf-16* mutants. These results suggest that age mutations (*age-1* and *daf-2* mutations) influence learning behavior in several ways.

Transcriptome analysis using adult *C. elegans* revealed IIS/FOXO and CREB transcriptional targets required specifically for memory and their tissue-specific expressions (Lakhina et al., 2015; Kaletsky et al., 2018). A nematode CREB homolog, *crh-1*, is required for long-term memory, but not for learning or short-term memory. The molecular mechanisms involving CREB were clarified in studies of positive butanone associative learning in *C. elegans* (Kauffman et al., 2011). Nematodes integrate the signals of butanone and food to enhance chemotaxis to butanone (Torayama et al., 2007). Expression of *crh-1* decreases with age and its expression and activation correlate with memory performance. The relationship between this *crh-1* pathway and the IIS pathway, however, is intricate. Insulin receptor *daf-2* mutants exhibit improved memory capacity in early adulthood and maintain learning ability with age. On the other hand, the *eat-2* mutant, which is a model of dietary restriction, exhibits impaired long-term memory in young adulthood and this low capability level is maintained with age. That is, rather than changes in the expression and activation of learning-related genes, it is a change in the expression and activation of *crh-1* that influences the IIS pathway and dietary restriction pathway in the decreased learning and memory observed with aging (Kauffman et al., 2010).

## IIS and Memory in *Lymnaea stagnalis*

Although the molecular tools available for use in gastropod mollusks are not as advanced as those for application in *Drosophila* and *C. elegans*, the contribution of the studies using mollusks to the elucidation of learning and memory mechanisms is significant (Kandel, 2001; Alkon et al., 2005). For example, *Aplysia* and *Limax* are well-known to be capable of associative learning (Yamanaka et al., 2021; Momohara et al., 2022). Furthermore, the pond snail *Lymnaea stagnalis* can be classically or operantly conditioned for many different types of behaviors (e.g., feeding and aerial respiratory behaviors; Sunada et al., 2017a; Crossley et al., 2019; Fodor et al., 2020; Itoh et al., 2021; Pirger et al., 2021; Rivi et al., 2021a, b). As described in the “Introduction” Section, ILP in *Lymnaea* was recognized as the first ILP with its DNA sequence revealed in invertebrates (Smit et al., 1988; Thorpe and Duve, 1988), leading to extensive studies of the relationship between insulin function and memory formation in *Lymnaea* (Pirger et al., 2014; Kojima et al., 2015; Benjamin and Kemenes, 2020).

**Figure 3:**
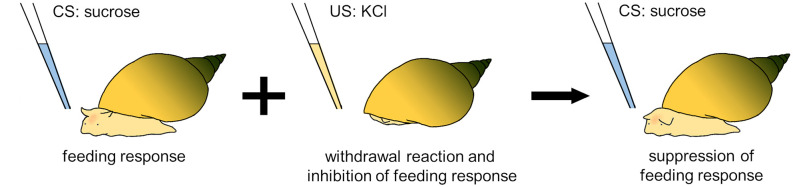
Training protocol for conditioned taste aversion (CTA) in *Lymnaea*. After a sucrose solution (CS) is paired with a KCl solution (US), the sucrose solution does not elicit a feeding response in snails.

In particular, *Lymnaea* has the ability to learn a conditioned taste aversion (CTA), which is formed by pairing the application of a sucrose solution as a conditioned stimulus (CS) and the application of a KCl solution or an electric shock as an unconditioned stimulus (US; Figure 3; Kojima et al., 1997; Nakai et al., 2020a). The sucrose solution evokes an innate feeding response, whereas the KCl solution or electric shock induces a withdrawal response. After repeated paired CS-US presentations, the sucrose solution (CS) no longer evokes the feeding response (Totani et al., 2020a). This CTA lasts longer than a month (Kojima et al., 1996). Because temporal changes in memory are generally classified into short-term memory, intermediate-term memory, and long-term memory (Rosenzweig et al., 1993), *Lymnaea* CTA is considered to be consolidated into long-term memory because of pharmacologic evidence requiring a *de novo* protein-synthesis processes (Nakai et al., 2020b).

Studies of the relationship between ILPs and memory in *Lymnaea* began with Azami’s work in 2006, which demonstrated that the expression of some genes was upregulated or downregulated following the formation of CTA and its long-term memory (Azami et al., 2006). In particular, upregulation of MIPs was observed in the CNS isolated from snails exhibiting CTA. Murakami et al. (2013a, b) then hypothesized that MIPs play an important role in altering the activity of feeding neural circuits that result in CTA (Hatakeyama et al., 2013). Applying MIPs or mammalian insulin to the isolated CNS induces long-lasting changes in synaptic efficacy (i.e., enhancement) between a key neuron for CTA (the cerebral giant cell: CGC) and a follower neuron (the B1 motor neuron). The B1 motor neuron innervates the salivary gland and plays a role in radula protraction (McCrohan and Benjamin, 1980a, b; Straub and Benjamin, 2001). These synaptic efficacy changes correlate with the consolidation of CTA into long-term memory in the snail CNS. This synaptic enhancement is blocked by the application of a human insulin receptor antibody, which is considered to block the binding between insulin (or MIPs) and human insulin receptors (or MIP receptors; Taylor et al., 1987). Injection of this human insulin receptor antibody into the snail abdomen before CTA learning blocks long-term memory formation, but not learning (Murakami et al., 2013a). A difference between the neurons critical for learning and those critical for long-term memory was also found in a CGC ablation study (Sunada et al., 2017b). The somata of the CGCs are not necessary for learning acquisition, whereas theyare necessary for long-term memory formation. Thus, both MIPs and the CGC somata play key roles in the memory consolidation of CTA.

Peptide purification of MIP I–III and V and the additional finding of a MIP VII transcript indicate that five types of MIPs function in *Lymnaea* (Smit et al., 1991, 1993, 1996; Li et al., 1992a, b, c). Their expression is observed in the growth-controlling neuroendocrine light green cells (LGCs) and canopy cells of the cerebral ganglia (Meester et al., 1992; Smit et al., 1992, 1998; Hatakeyama et al., 2000). The cDNA structure of a putative tyrosine kinase receptor for MIPs has also been clarified (Roovers et al., 1995). Many of the typical insulin receptor features, including a cysteine-rich domain, a single transmembrane domain, and two cytoplasmic domains for trans-phosphorylation to induce tyrosine kinase activity, are also conserved in the predicted 1607-amino acid protein in *Lymnaea*. Extensive screening of cDNA and genomic libraries together with Southern blot analyses have revealed the presence of a single putative MIP receptor gene and suggest that different MIPs bind to the same receptor (Smit et al., 1996).

As described earlier, MIPs reduce the hemolymph glucose concentration in *Lymnaea* (Mita et al., 2014a), and thus the relationship among the actions of MIPs, nutritional status, and CTA learning ability should be examined (Totani et al., 2020b). Mild starvation (i.e., 1-day food deprivation) results in the best learning and memory for CTA, whereas heavy starvation (i.e., 5-day food deprivation) prevents snails from learning or remembering (Aonuma et al., 2018a). Injecting the snails with mammalian insulin to reduce the hemolymph glucose concentration results in better learning and memory in 5-day food-deprived snails, but injecting glucose into 5-day food-deprived snails does not alter their inability to learn and remember (Mita et al., 2014a, b). On the basis of these observations, the “insulin spike hypothesis” (i.e., a rise in the insulin concentration in the CNS of snails) was proposed for the formation of CTA and its long-term memory (Mita et al., 2014a, b; Kojima et al., 2015).

Evidence to support this hypothesis was obtained in 2020 (Totani et al., 2020c). As mentioned above, 1-day food-deprived snails exhibit the best CTA learning and memory, whereas 5-day food-deprived snails do not express good memory. CTA and its long-term memory, however, are indeed formed in 5-day food-deprived snails, but memory recall for the CTA is prevented by the effects of food deprivation. Long-term memory recall in 5-day food-deprived snails is expressed following 7 days of feeding and then 1 day of food deprivation. Totani et al. (2020c) revealed that this 1 day of food deprivation before the memory test in snails increased the mRNA levels of MIP II, a major MIP, in the CNS. Instead of this 1-day food deprivation, injection of insulin into snails activates CTA neurons and mimics the food deprivation state before a memory test. Together, these results suggest that a spike in insulin release recapitulates the optimal internal state for long-term memory recall following CTA training in snails (Totani et al., 2020c).

The neurocircuit involved in CTA in *Lymnaea* has been examined from the viewpoint of some neurotransmitters, such as monoamines (Yamanaka et al., 2000; Kawai et al., 2011), and the relationship between monoamines and insulin in CTA learning ability has been examined (Aonuma et al., 2016, 2017; Totani et al., 2019). One monoamine, serotonin (5-hydroxytryptamin; 5-HT), is known to be involved in decision-making in *Lymnaea* (Aonuma et al., 2020). Although the snails with 1-day food deprivation exhibit the best learning and memory for CTA, immersion of 1-day food-deprived snails in 5-HT impairs CTA learning and memory by increasing the 5-HT content. Furthermore, injection of mammalian insulin into these snails reverses this impairment (Aonuma et al., 2018b). That is, insulin rescues the CTA deficit, which may be due to a decrease in the 5-HT content in the CNS. Totani et al. (2022) further examined why CTA learning ability is worse in 5-day food-deprived snails than in 1-day food-deprived snails and how the CNS 5-HT concentration increases in 5-day food-deprived snails (returns to basal levels). They measured the concentration of tryptophan (i.e., 5-HT precursor) in the hemolymph and CNS, and demonstrated that the CNS tryptophan concentration was higher in 5-day food-deprived snails than in 1-day food-deprived snails, whereas the hemolymph tryptophan concentration was not affected by the duration of food deprivation. This finding suggests the existence of a mediator of the CNS tryptophan concentration independent of food deprivation. This mediator was then identified as “autophagic flux” in the CNS under different food deprivation conditions. The tryptophan concentration in the hemolymph and the autophagic flux in the CNS cooperatively regulate CTA learning ability affected by different durations of food deprivation.

## Perspective and Conclusion

**Figure 4:**
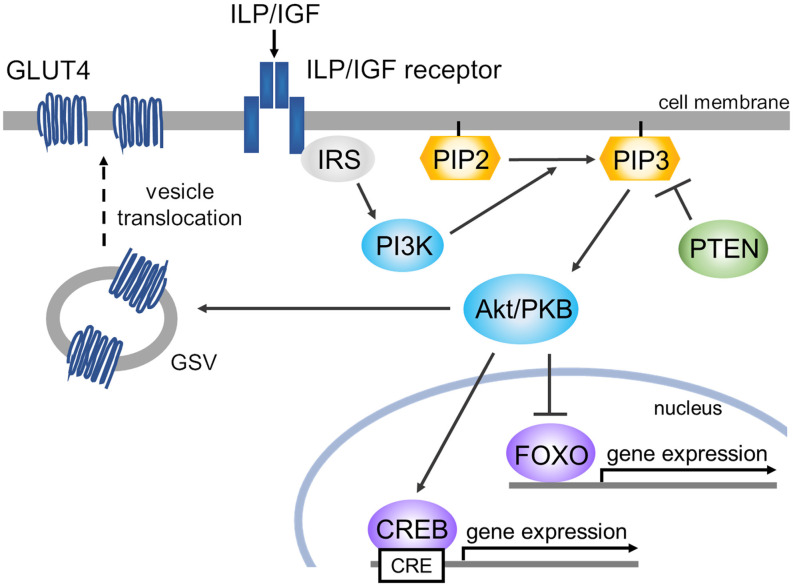
Scheme of deduced ILP/IGF signaling cascades for memory enhancement. There are three main pathways: (1) Akt/PKB phosphorylates CREB (e.g., CREB1 in *Lymnaea*), resulting in gene expression. (2) Akt/PKB phosphorylates FOXO, inducing its secession from DNA. (3) Akt translocates GSV to the membrane, resulting in the expression of GLUT4. The ILP/IGF receptors are called InR in *Drosophila*, DAF-2/IGFR (insulin/IGF-1 transmembrane receptor) in *C. elegans*, and MIPR in *Lymnaea*. Abbreviations: Akt/PKB, Akt/protein kinase B; CREB, cAMP response element-binding protein; FOXO, forkhead box protein O; GLUT4, glucose transporter isoform 4; GSV, GLUT4 storage vesicle; IRS, insulin receptor substrate; PI3K, phosphoinositide-3-kinase; PIP2, phosphatidylinositol 4,5-bisphosphate; PIP3, phosphatidylinositol 3,4,5-trisphosphate; PTEN, phosphatase and tensin homolog deleted on chromosome 10 (i.e., PIP3 phosphatase).

These findings together provide a perspective of the deduced scheme for involvement of the insulin pathway in cognitive function in invertebrates (Figure 4). We note two kinds of transcription factors in IIS: CREB and FOXO, which are well analyzed in the above-mentioned three species of invertebrates. ILP/IGF binding to its receptor tyrosine kinase drives the phosphoinositide-3-kinase (PI3K)-Akt/protein kinase B (PKB) pathway (Belfiore et al., 2009; Hay, 2011). The PI3K-Akt/PKB pathway regulates CREB for gene expression (Du and Montminy, 1998; Thiel et al., 2021). In *Lymnaea*, activation of CREB plays a critical role in the formation of long-term memory (Ribeiro et al., 2003; Sadamoto et al., 2004, 2010), and at the mRNA level, the amount of suppressor CREB (e.g., *Lymnaea* CREB2) is more abundant than the amount of activator CREB (e.g., *Lymnaea* CREB1; Wagatsuma et al., 2005). The other transcription factor, FOXO, is “inactivated” by the PI3K-Akt/PKB pathway (Lin et al., 2001; Hay, 2011). FOXO, however, is involved in learning and memory formation in a starved state (Nagashima et al., 2019). That is, the function of FOXO remains a mystery, and likely has two different roles. Akt translocates GLUT4 storage vesicles (GSVs) to the membrane, resulting in the expression of GLUT4. GLUT4 is glucose transporter isoform 4 and is suggested to be involved in memory formation (Grillo et al., 2009).

In conclusion, here we have reviewed the relationship between insulin and memory in *Drosophila*, *C. elegans*, and *Lymnaea*. We propose that the mechanisms underlying the involvement of insulin in cognition in invertebrates occur across phyla, including humans, although findings of the molecular mechanisms in mammals are scant. It is important to note that learning ability depends on the state of starvation in invertebrates. Short-term fasting in mammals also enhances cognition, including memory consolidation (Dias et al., 2021), whereas obesity impairs cognition and increases the risk of dementia in humans (Shinjyo and Kita, 2021). Extensive studies using invertebrates can provide important insight into the advantages of short-term fasting in humans. In addition, by using invertebrates, it is easier to investigate the complex interplay between the specific roles of insulin and not only cognitive abilities but also longevity and stress tolerance. This insight is also important for translational medical studies.

## Author Contributions

EI conceptualized the study. JN drew the figures. All authors (JN, NC, KF, YT, DH, VED, and EI) participated in the study design and manuscript preparation, and approved the submitted manuscript and final manuscript. All authors contributed to the article and approved the submitted version.

## Conflict of Interest

The authors declare that the research was conducted in the absence of any commercial or financial relationships that could be construed as a potential conflict of interest.

## Publisher’s Note

All claims expressed in this article are solely those of the authors and do not necessarily represent those of their affiliated organizations, or those of the publisher, the editors and the reviewers. Any product that may be evaluated in this article, or claim that may be made by its manufacturer, is not guaranteed or endorsed by the publisher.
